# Hard‐Wired Solid‐State Bioelectronic Micropore Devices: Permanent Metal‐Protein‐Metal Junction Proof‐of‐Concept

**DOI:** 10.1002/smll.202506560

**Published:** 2025-10-19

**Authors:** Sudipta Bera, Eran Mishuk, Ping'an Li, Sourav Das, Sigal Keshet, Sharon Garusi, Leonid Tunik, Eran Edri, Yoram Selzer, Israel Pecht, Ayelet Vilan, Mordechai Sheves, David Cahen

**Affiliations:** ^1^ Department of Molecular Chemistry and Materials Science Weizmann Institute of Science Rehovot 7610001 Israel; ^2^ Department of Chemical Research Support Weizmann Institute of Science Rehovot 7610001 Israel; ^3^ Department of Immunology and Regenerative Biology Weizmann Institute of Science Rehovot 7610001 Israel; ^4^ Department of Chemical and Biological Physics Weizmann Institute of Sciences Rehovot 7610001 Israel; ^5^ Department of Chemical Engineering Ben Gurion University of the Negev Beer‐Sheva 8410501 Israel; ^6^ Department of Chemical Physics School of Chemistry Tel Aviv University Tel Aviv 69978 Israel

**Keywords:** biomolecular electronics, evaporated top‐contact, impedance, micropore device, permanent contact, protein thin film

## Abstract

The design, fabrication, and application of robust metal/protein/metal junctions are presented with ultrathin (≈20 nm) protein films demonstrating long‐term stability in ambient conditions and preserving their electron transport behavior also at ≈10 K. These junctions establish a reliable platform with a permanent contact configuration, where the confined protein layer retains its functional activity after metal contact evaporation on the protein. A bottom‐up micropore device (MpD) fabrication strategy is used and optimized to ensure reproducibility. The sub‐nanometer roughness of the bottom electrode is preserved within the micropore, enabling uniform protein layer deposition and film formation. In the MpD structures, protein layers are integrated between Au‐covered substrates and an e‐beam evaporated Pd contacts. Depositing multi‐layered protein films allows for defining film widths, as tested by the atomic force microscopy (AFM)‐based scratching technique. The films are composed of human serum albumin (HSA) and bacteriorhodopsin (bR). Pd's preferred 2D growth minimized metal penetration and short circuits. Impedance phase response analysis shows that ≈60% of the junctions are functional ones, demonstrating the effectiveness of the fabrication approach. These protein‐based MpD junctions provide a basis for future stable platforms for electron transport studies of bio‐ and other soft materials.

## Graphical abstract

A bottom‐up way to fabricate precisely engineered micropore solid‐state metal substrate/protein film / evaporated metal structures is shown to be a versatile platform for systematic electron transport measurements. As protein function is preserved and the junctions are thermally stable, down to 10K, such junctions can be useful for bioelectronic applications.

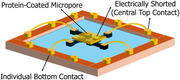

## 1 Introduction

The study of electronic transport in biomolecules, like proteins, is not only fascinating per se but also necessary for possible applications in true bioelectronics. However, a critical bottleneck persists: the lack of reliable and durable metal/biomolecule/metal electrical junctions, which are essential for probing, understanding, and controlling electron flow across molecules. Most research to date uses self‐assembled protein monolayers deposited on a conductive substrate as electrode contact.^[^ ^1^, ^2^ ^]^ However, as *mono*layers of proteins are ultra‐thin (≤5–10 nm), relying on them leads to the nearly exclusive use of vertical junctions. Implicit in that choice is the problem of how to contact such films. Specifically, depositing top electrodes directly onto protein layers, without protective interlayers, can lead to damage or poor contact with the protein, to poor reproducibility, lack of device stability, and combinations of these.

Part of those issues result from the need for ultra‐smooth bottom electrodes to achieve optimal and reproducible substrate (  = contact) coverage, which requires minimizing surface roughness, as even minor imperfections can disrupt continuous protein layer assembly. Still, even with optimally packed protein monolayers, interstitial voids are unavoidable because of the shapes of these biomolecules.^[^ ^3^ ^]^ Voids expose the underlying substrate, creating pathways for electrical leakage and short‐circuits. These challenges highlight the need for new approaches to junction fabrication that balance durability, precision, and scalability to pave the way for breakthroughs in both fundamental science and practical applications of bioelectronics. Until now, various approaches, including mechanically applied contacts such as floating‐on ready‐made metal pads,^[^ ^4^, ^5^ ^]^ dielectrophoretic trapping of Au nanowires,^[^ ^6^, ^7^ ^]^ hanging Hg drops,^[^ ^8^, ^9^ ^]^ and Ga‐In eutectic (EGaIn) cones or microfluidic arrays,^[^ ^10^, ^11^ ^]^ have been employed.

Mechanical placement of contacts often raises concerns regarding contact quality and reproducibility, especially control over the electrically active contact area (as a fraction of the geometric one).^[^ ^12^ ^]^ Reducing the dimensions of one of the electrodes decreases the last problem. A special type of mechanical contact is the use of an electrically conducting AFM tip for conductive atomic force microscopy (C‐AFM) to make temporary metal/molecule/metal junctions, where reproducibility remains a challenge ^[^ ^13^ ^]^ as maintaining known consistent contact areas across multiple measurements is difficult. C‐AFM can probe transport in molecular junctions formed within specially designed silica nanopores.^[^ ^14^ ^]^ Molecular break junctions (MBJ) provide another route for creating (lateral) metal/molecule/metal junctions, but they are limited to one or very few molecules, and as the molecules get longer, the signal decreases. In general, MBJ exhibits a very broad statistical distribution, necessitating extensive data collection for reliable characterization, which makes it a useful tool for fundamental research.^[^ ^15^ ^]^ Hitherto, only ferritin, a unique protein, could be studied in this way.^[^ ^16^ ^]^

In earlier studies of protein junctions with evaporated top electrodes, electrical shorts were avoided by adding small molecules that pack well to form a dense layer between the proteins and the top electrode.^[^ ^17^ ^]^ However, this approach complicates addressing the intrinsic protein transport properties due to the intervening molecular layer. Earlier, we succeeded in evaporating Pb onto well‐packed organic molecular monolayers, without damaging the terminal exposed groups of the organic.^[^ ^18^, ^19^ ^]^ Recently, we successfully evaporated a carbon‐based top contact onto a protein layer on a Au substrate.^[^ ^20^ ^]^ The disadvantage of this type of protein junction is the relatively high resistance of the carbon contact that limits electron transport (ETp) measurements, particularly for thicker protein junctions. Li and Selzer developed nanopore devices evaporating Bi as the top electrode,^[^ ^21^, ^22^ ^]^ successfully forming junctions with the small protein Azurin.^[^ ^22^ ^]^ The small pore dimension (100 × 100 nm^2^) dictates a small protein junction area, which is a challenge for in situ characterization of layer quality by AFM imaging and limits transport studies as a function of protein film thickness (because of increasing film resistance).

Inspired by the work of Li and Selzer,^[^ ^21^ ^]^ we fabricated and characterized micropore devices (MpDs) for stable, non‐shorted, and area‐controlled metal/protein/metal junctions with minimal contact resistance.^[^ ^23^ ^]^ Here, the term micropore follows the convention, where “micro‐” indicates micrometer‐scale features (as in microchannels, micropillars, microneedles), rather than the IUPAC pore‐size classification for bulk porous media (see also ref.[24]). These MpDs enable ETp studies across thicker protein junctions than the nanopore ones, while maintaining exceptional stability in cryogenic conditions with permanent contacts. Notably, the MpD‐based protein junctions exhibit minimal temperature dependence in ETp, aligning with observations for other protein junctions, for films up to 60 nm thick.^[^ ^1^, ^2^, ^5^, ^12^, ^25^, ^26^ ^]^ To demonstrate the potential of MpDs, we use HSA and bR as model proteins, revealing distinct current responses for each. Optically transparent glass/bR/Pd structures with evaporated Pd exhibit a well‐defined photocycle response, confirming the retention of this protein's conformation in the MpD. Additional photo‐elastic modulated infrared reflection absorption spectroscopy (PEM‐IRRAS) analysis broadly indicates that the protein's structural conformation is largely retained, even in the presence of the top‐evaporated Pd layer. Junction reliability is further validated through high‐frequency impedance phase analysis.

## 2 Results and Discussion

**Scheme 1:**
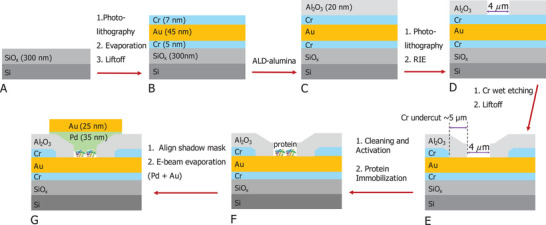
A–D) Micropore device fabrication process scheme illustrating building up and exposure of the bottom electrode structure, with C) addition of alumina insulation layer by ALD, D) patterning and etching of alumina layer to uncover the bottom Cr etch stopping layer, and E) Cr wet etch to uncover the Au layer that will serve as bottom and as area‐defining electrode contact. This process leads to a large undercut, causing the 20 nm alumina layer to collapse on the Au (see Section 2.3 in the main text) F) activation of the Au surface and immobilization of the protein G) final steps of shadow mask alignment and deposition of the top contact.

**Scheme 2:**
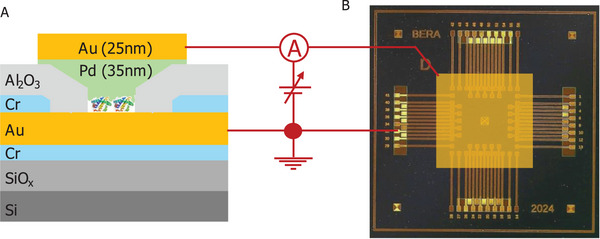
Device configuration and biasing for electrical measurements: A) Cross‐sectional scheme of the sandwiched protein‐based MpD, and B) image of an actual MpD chip with 56 integrated devices and the (Pd/Au) evaporated central common top electrode (yellowish square).

The MpDs are multilayered structures with a micro‐electrode‐bound protein film, sandwiched between two metal contacts (**Scheme** **1**). Each MpD chip comprises 16/56 identical MpDs, packed within a 5 × 5 mm^2^ area. The central 2 × 2 mm^2^ region is the active zone of the chip, where the micropores are situated, as illustrated in **Scheme** **2** and Figure S1 (Supporting Information). A detailed fabrication procedure is given in the experimental section and in the Supporting Information.

### 2.1 Optimization of the Bottom Electrode Quality

The surface quality of the bottom electrode is critical because the packing quality of the multilayers, and therefore that of the resulting devices, depends on the smoothness and homogeneity of the first adsorbed layer. Assuring this quality for the bottom electrode surface is challenging due to multiple deposition and etching steps during fabrication. In the standard bottom electrode configuration (Si/SiO_x_/Cr/Au/Al_2_O_3_), the lack of a protective layer during reactive ion etching (RIE) to remove the alumina layer was found to be problematic. During RIE, both the alumina and the underlying Au layer were etched, as the etching rate of Au exceeds that of the ALD‐grown alumina (Figure S2, Supporting Information). Attempts to mitigate this by reducing etching time resulted in either residual alumina within the micropores or root‐mean‐square (RMS) roughness > 3 nm of the exposed Au layer. To address this issue, a protective Cr layer was introduced atop of the Au layer, creating an enhanced electrode stack (Si/SiO_x_/Cr/Au/*Cr*/Al_2_O_3_) (see Scheme 1). Control experiments demonstrated that the Cr layer was not etched during a 400 s, which is the optimized RIE time for alumina (Figure S3, Supporting Information). Rather than being etched, the micropore area appears slightly elevated with a rough surface following plasma treatment. This plasma‐treated Cr layer could be completely removed through wet‐Cr etching. The chromium etchant has high selectivity, effectively removing the Cr without affecting the underlying Au layer. About 30 s etching removes all the (exposed) protective Cr, leaving the underneath smooth Au surface (Figure S4, Supporting Information). Thus, the 7 nm Cr protective layer prevented unwanted Au etching during RIE, while allowing exposure of the original smooth Au surface through selective wet etching, ensuring the bottom electrode's quality, especially its minimal roughness. This procedure opened the way to producing high‐performance, multi‐layered MpD configurations.

### 2.2 Rationale for Choosing ALD‐Alumina

To create an electrical micropore junction, it is essential to coat the conductive electrode with an insulating layer. We selected alumina for several reasons. Alumina can be prepared in a highly controlled manner by atomic layer deposition (ALD), and its ALD precursors are cost‐effective and chemically stable over a wide pH range. ALD‐grown alumina ensures uniform layer coverage (Figure S5, Supporting Information) with minimal pinhole formation, in contrast to plasma‐enhanced chemical vapor deposition (PECVD), sputtering, or E‐beam‐based physical vapor deposition, which can introduce defects.^[^ ^27^ ^]^ Importantly, the resulting oxide layer is highly resistive (≈10^14^ Ω·m). For this study, a 20 nm alumina layer was chosen, as it provides an effective, complete coverage without pinholes of an insulating coating over the conductive bottom electrodes. The 20 nm alumina layer suffices to study protein multilayers while ensuring complete electrical insulation integrity; no current can be measured (noise level 200 fA) through the 20 nm alumina layer over the ±3 V voltage sweep.

### 2.3 Collapsing Alumina at Pore Boundaries and Fabrication Limits

**Figure 1:**
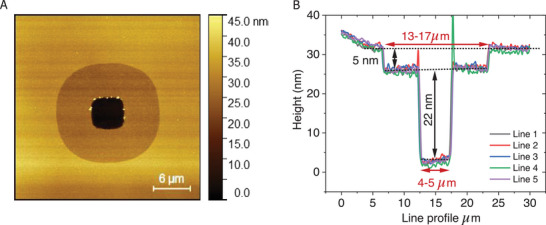
A) AFM topography of the micropore electrode, showing the top alumina layer exhibiting shrinkage around the micropore region, visible as a light brown, donut‐shaped structure surrounding the central pore. This shrinkage is attributed to the undercut chromium layer. The scattered yellow spots at the pore boundary indicate residual alumina waste accumulated from the dry etching process (see Experimental Section‐Micropore Fabrication at the Bottom Electrode). B) Cross‐sectional profile of the micropore area, where the long horizontal red double‐headed arrow highlights the shrinkage region. The short black double‐headed arrow denotes a 5 ± 1 nm thick shrinkage alumina layer, and the long vertical black double‐headed arrow indicates a net pore depth of ≈22 ± 2 nm. The micropore dimensions are reported as mean ± standard deviation (SD), based on a sample size of *n* = 10.

Wet etching of the protective Cr layer resulted in an unavoidable undercut near the micropore region, leading to observable device collapse up to 7 nm in height (Scheme 1E, **Figure** **1**) at the micropore boundary, which, though, leaves the pore area (≈20 *µ*m^2^) with the exposed Au‐bottom electrode unaffected. This phenomenon occurs because the voids created by the removal of the protective Cr layer (between the bottom Au and alumina) are rapidly filled by the alumina, which collapses on and strongly adheres to the underlying exposed Au layer. AFM images distinctly revealed the shrinkage effect around the micropore area (Figure 1). This extra alumina cover at the shrunken part of the pore boundary shows increased fragility under certain conditions. Treatments such as O‐plasma or base piranha caused further weakening, but it remains intact even after 30 min of ultrasonic bath sonication (before base‐piranha treatment).

The exposed Au surface must be activated for efficient protein or linker binding to ensure dense molecular coverage. However, O‐plasma treatment was found to increase the roughness of the bottom Au electrode (Figure S6, Supporting Information). Comparative analysis revealed that *mild base piranha* was more effective than the O‐plasma treatment (Figure S7, Supporting Information). Base piranha provided effective surface cleaning and facilitated activating the Au surface while minimizing surface roughness (Figure S7, Supporting Information) for subsequent linker or protein immobilization.

### 2.4 Selection of the Top Electrode

E‐beam evaporated palladium (Pd) was used as the top contact for the protein junctions. Unlike metals such as Au, Pd predominantly exhibits two‐dimensional surface growth during evaporation, which minimizes the likelihood of forming short‐circuited junctions through filament formation.^[^ ^28^ ^]^ The evaporation protocol (detailed in **Experimental Section‐Top Electrode Deposition**) with an operational electron gun (low energy relative to thermal deposition), continuous sample stage cooling, and angled semi‐indirect evaporation helps to preserve the integrity of the protein layer and prevents electrical shorting. Due to the susceptibility of Pd to oxidation, a protective layer of gold (25 nm) was deposited onto the freshly evaporated Pd layer without breaking the vacuum.

### 2.5 Criteria for Junction Selection and Electrical Transport Characterization

Protein monolayer‐based MpD preparation frequently yields short‐circuited junctions. This problem decreases as protein layers are added on top of the monolayer, allowing reasonable yields (≈60%) of functional junctions of protein layers that are over 15 nm thick. The reason is that the first protein layers that self‐assemble on the bottom electrode leave substantial voids (Figure S8, Supporting Information). During evaporation of the top electrode, filamentous growth can occur in these voids, leading to electrically shorted junctions. The larger the protein size, in terms of its footprint on the substrate, the lower its packing density, i.e., the more of the bottom substrate remains exposed, i.e., the higher the probability of shorts. Elsewhere, we report that consistently transport‐active (non‐shorted) junctions were achieved using the good coverage quality of protein bilayers or thicker multilayers^[^ ^23^ ^]^ to block direct exposure to the bottom electrode.

#### 2.5.1 Insight into Protein‐Coated MpD

**Figure 2:**
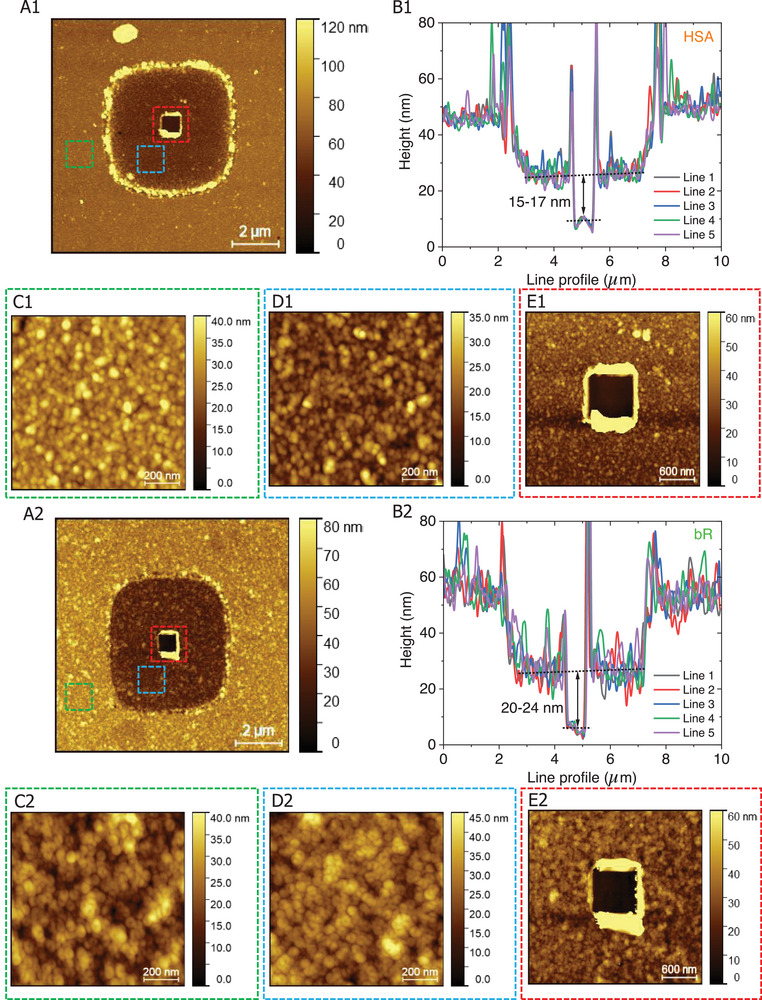
The representative AFM scratched image of A1) HSA tetra‐layer and A2) bR triple‐bilayer at the center of the micropore (red dotted square) on MpD. Corresponding cross‐sectional line profile, indicating protein layer thickness B1) 16 ± 1 nm (for HSA) and B2) 22 ± 2 nm (for bR) [mean ± SD; *n* = 5]. The protein layer spans both the micropore and the surrounding alumina surface. AFM topography images of the C1) HSA and C2) bR layer on top of the alumina surface (represented as a GREEN dotted box), D1,D2) over the micropore on the Au bottom electrode (represented as a BLUE dotted box); E1,E2) at the micropore center region after scratching for HSA and bR respectively (represented as a RED dotted box). Colored dotted boxes highlight specific micropore regions, as indicated above.

We show and discuss results for two specific protein junctions in MpDs, containing 16 ± 1 nm HSA *tetra*layers (quadruple monolayers) and 22 ± 2 nm bR *triple* bilayers. The protein layer thickness was estimated from high contact force‐controlled AFM scratching experiments (see **Experimental Section‐AFM Characterization**) conducted in the micropore (**Figure** **2**). During protein layer preparation in MpDs, we used a separate witness Au‐substrate to monitor the growth of the protein layer by ellipsometry and found it to be consistent with AFM scratch‐derived thickness.^[^ ^5^, ^7^ ^]^ Since ellipsometry cannot be applied directly to micro‐pores, we prepared protein layers, in parallel, on flat Au substrates. These served as proxies, and ellipsometry results on them confirmed the AFM findings from the scratching experiment, validating the uniformity and reproducibility of the protein layers. In MpDs, protein layers are formed uniformly across the chip, covering both the exposed linker‐coated Au surface (for bR; HSA can bind directly without a linker)^[^ ^23^ ^]^ area in the micropores and the alumina surface (Figure 2). Given that alumina is positively charged^[^ ^29^ ^]^ at the pH used for protein self‐assembly (5.5–7), while the surfaces of both bR and HSA have a net negative charge, electrostatic (non‐specific) binding facilitates the formation on alumina of the first protein layer. After that, EDC‐activated covalent coupling‐based sequential layer‐by‐layer immobilization leads to protein multilayer formation over the entire chip, including the alumina surface, outside the micropore. Thus, the protein layer that is deposited always maintains a constant depth of the micropore (≈20 nm), relative to the rest of the device area, even after protein layer immobilization (Figures 1 and 2).

#### 2.5.2 Impedance‐Based Selection of Transport Active Junctions

An essential method for identifying the transport active state (non‐shorted with stable, reproducible I‐V response characteristics) of these protein junctions is impedance response signal analysis. Our experimental impedance data indicate that the transport active protein junctions (with measurable direct current (DC) current‐voltage (*I*–*V*) characteristics) can be modeled with a parallel resistor‐capacitor (R‐C) circuit, with both resistive and capacitive components.^[^ ^5^, ^23^ ^]^ These parameters can be extracted from the Nyquist plot, where both resistance and capacitance are evident from the impedance fitting based on the chosen equivalent circuit.^[^ ^30^ ^]^ The Bode plot supports this analysis: the impedance magnitude versus frequency teaches about resistive behavior, and the impedance phase versus frequency about the quality of the dielectric layer.

**Figure 3:**
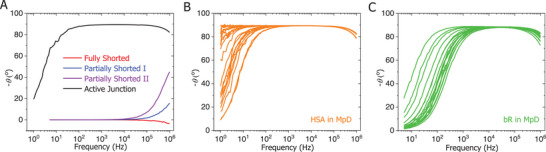
Bode plots of the impedance phase as a function of frequency for different protein junctions in MpD configuration. A) The characteristic high‐frequency phase response effectively distinguishes active protein junctions from partially or fully shorted junctions (representative), as indicated in the figure legend; Bode plots of stable transport active junctions of B) HSA tetra‐layer, C) bR triple bi‐layer (*n* = 20).

Here, we focus primarily on the capacitive characteristics of the protein layer to evaluate its performance as an effective dielectric medium between two terminal electrodes, specifically as a reporter for the presence of any filamentary metallic pathways. The specter of the formation of such filaments hangs over any junction with a vacuum evaporated top, which can lead to high leakage currents and degrade the capacitive behavior of the junction. Such degradation will directly influence the phase of the impedance. Thus, we use the phase versus frequency response as a diagnostic tool to distinguish well‐behaved, transport‐active protein junctions from defective ones. High‐frequency impedance phase analysis of complete devices confirmed protein film integrity in MpD junctions, ensuring reliable transport through active junctions. In a parallel resistor–capacitor (R–C) circuit (cf. ref^[^ ^5^ ^]^), at high frequencies (≈10 kHz or above), the AC current fully passes through the capacitor component in protein junctions (without dielectric leak),^[^ ^23^ ^]^ producing an alternative current (AC) phase difference of 90°. Such a phase angle tells us that the DC current that we measured in protein‐based MpD junctions is unlikely to be affected by shorts (see **Figure** **3**). In contrast, a fully shorted junction exhibits an ≈0° phase shift, where the unwanted metal filament grown by the top electrode evaporation electrically connects to the bottom electrode, bypassing the protein layer and eliminating the parallel R‐C nature of the original configuration (Figure 3A). Partially shorted junctions display a high‐frequency phase shift between 0° and 90°, indicating some dielectric leakage. Such junctions (high frequency θ well below 90°) may have a significant filamentary path that formed within the solid‐state protein matrix, reaching close to the bottom electrode but remaining non‐shorted. Practically, this translates into a much thinner junction than expected from the directly measured film width. These junctions are highly unstable and often evolve into fully shorted states after a few DC voltage sweeps.^[^ ^7^ ^]^ Therefore, a distinct phase response (as a part of the Bode plot) enables selecting the functional protein junctions (Figure 3), ^[^ ^5^ ^]^ and only impedance‐qualified MpD junctions (θ ≈ 90° above 10 kHz) were used for electron transport studies. Approximately 60% of the multi‐layered protein junctions were not‐shorted, with over 40% demonstrating stable performance over the full experimental voltage range (±0.1 V), i.e., reproducible back and forth voltage sweeps, making them suitable for further transport measurements.

#### 2.5.3 Junction *J*–*V* Response

**Figure 4:**
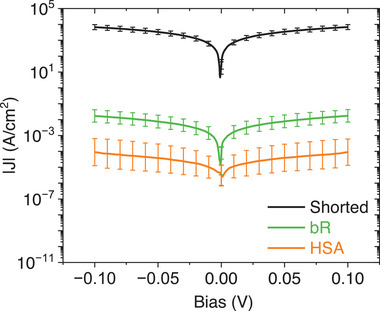
Semi‐logarithmic plot of the averaged current density *(J)*, versus applied voltage bias for more than 20 junctions with a triple bR‐bilayer and HSA‐tetralayer in the MpD configuration. The plot includes the response of shorted junctions and indicates the mean values with error bars representing the standard deviations (mean ± SD; *n* = 20–25).

DC measurements provided junction currents characteristic of HSA or of bR junctions (**Figure** **4**). The variations in junction current density (*J*) (mean ± SD) are sufficiently small so that the junctions with the two types of proteins can be clearly distinguished (Figure 4). These protein junction currents are several (6‐8) orders of magnitude lower than those observed in shorted junctions (Figure 4). The obtained average junction current density indicates that the thicker bR junctions transport electrons more efficiently than the thinner HSA junctions,^[^ ^23^ ^]^ consistent with previous work^[^ ^31^ ^]^ using different contacts and junction configurations.

### 2.6 Stability and Functional Activity of Protein Junctions in MpD Configuration

#### 2.6.1 *J*–*V* Response Reproducibility

**Figure 5:**
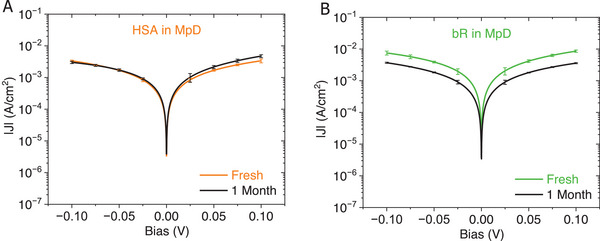
*J* versus *V* characteristics of protein junctions in the MpD configuration, measured both fresh junctions immediately after fabrication and after one month. The representative protein‐multilayer junctions: A) HSA tetra‐layer junction. B) bR triple‐bilayer junction. For each representative protein junction, the *J*–*V* characteristics were obtained by averaging two consecutive voltage sweeps. The results are presented as mean ± 2 × SD (*n* = 2), reflecting the 96% confidence interval range.

Fabricating stable protein junctions remains a significant challenge. Most of the junctions can survive over a week with a consistent current response. But over a more extended period, only a few survive, which shows negligible change in current‐voltage response even after 1 month (see **Figure** **5**), consistent with previous reports that show it is possible to have solid‐state protein junctions that are stable over days to weeks.^[^ ^32^ ^]^

#### 2.6.2 Junction Robustness in Permanent Contact Configuration

**Figure 6:**
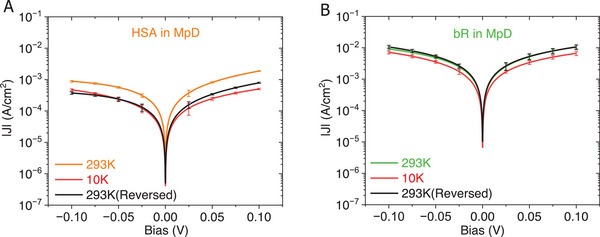
Representative *J* versus *V* characteristics of protein junctions in MpD configuration measured at three different temperatures: room temperature (293 K), 10 K, and after returning to room temperature. The representative protein‐multilayer junctions: A) HSA tetra‐layer junction. B) bR triple‐bilayer junction. Here, the *J–V* characteristics were obtained by averaging two consecutive voltage sweeps, which is represented as mean ± 2 × SD (*n* = 2), corresponding to the 96% confidence interval.

Temperature‐dependent transport behavior is crucial for understanding electron transport mechanisms. Many protein junctions have configurational constraints that limit their compatibility with cryogenic setups. However, MpD junctions are robust, providing consistent electrical transport characteristics in both the hitherto used probe station‐based measurements and as hard‐wired, bonded ones for use in sub‐liquid N_2_ temperature cryogenic setups. The experimental setup and methodology are given in Section S1 (Supporting Information). Throughout the temperature cycle (293 K ↔ 10 K), the junction (MpD) current response remains stable, showing minimal temperature dependence, i.e., variations within a factor of two (see **Figure** **6**). Notably, the ≈20 nm‐wide protein junction displayed near temperature independence with activation energy below kT@RT (<26 meV), a result that challenges existing transport mechanisms. This phenomenon is explored in greater detail in an upcoming study.

Lack of temperature dependence has been reported for ultra‐thin (1.5–4 nm) protein junctions^[^ ^1^, ^2^ ^]^ and was attributed to quantum tunneling. Surprisingly, we observed similar behavior as also for thicker (5–9 nm) junctions across multiple labs,^[^ ^12^ ^]^ and more recently, even up to ≈60 nm,^[^ ^5^ ^]^ where neither conventional tunneling nor thermally activated hopping can explain the results. While models can fit at least part of the data.^[^ ^33^ ^]^ At this stage, there is no mechanism to explain these surprising findings.

#### 2.6.3 Maintaining Protein Functional Stability under the Evaporated Contact

Extensive control experiments and stability tests confirm that protein junctions in the MpD configuration exhibit exceptional stability. However, uncertainty remains regarding their functional activity in this configuration. Most solid‐state protein junctions are fabricated using soft‐contact methods,^[^ ^1^, ^5^, ^34^ ^]^ where the surface characterization studies suggest that proteins maintain their original (solution) conformation.^[^ ^20^, ^31^ ^]^ In contrast, the MpD configuration has an evaporated metal top contact, which might, in principle, denature the proteins, even just partially. To address this, we used the fact that bR is a photoactive protein. In bR a photochemical process occurs, as the retinal moiety inside the bR undergoes a photo‐induced cis‐trans double bond isomerization with concomitant changes in the peptide matrix, surrounding the retinal. Upon exposure to > 500 nm light, bR in solution experiences a photocycle consisting of several intermediates, including one called “M”, which absorbs at ≈410 nm and, thermally, or upon exciting at that short wavelength, decays to the original pigment in ≈20 ms.^[^ ^20^, ^35^ ^]^

**Figure 7:**
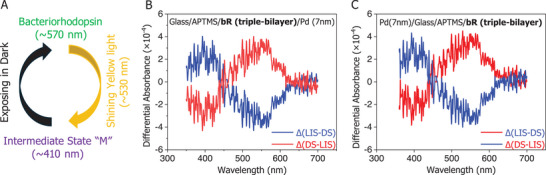
A) Schematic representation of the bR photocycle with corresponding UV–vis scans under dark–light–dark conditions (see Section S2.4, Supporting Information). The Light‐Induced Spectrum (LIS) and Dark Spectrum (DS) are shown as differential spectra. Upon illumination, the appearance of the characteristic “M” intermediate is evidenced by a positive peak at ≈410 nm (blue‐spectra) in the ∆(LIS–DS) spectrum. Under dark conditions, bR exhibits its characteristic ≈570 nm peak, seen as a positive peak (red‐spectra) in the ∆(DS–LIS) spectrum. B, C) display the combined differential LIS and DS spectra for devices with an evaporated top Pd contact and backside Pd contact, respectively, serving as a control. Details of the transparent junction configuration are provided in the figure legend.

The protein layer trapped in the MpD is not suitable for testing photoactivity using either transmission or reflection modes. Instead, we designed an optically transparent sandwiched bR junction in the configuration glass/APTMS/bR (triple‐bilayer)/Pd (7 nm). The experimental setup and preparation details are provided in Section S2 (Supporting Information). Upon illumination with yellow light, the bR junction exhibited the expected optical response, with reversible appearance and disappearance of the ≈410 nm M‐intermediate peak (see **Figure** **7**).

With this setup, we did measurements to determine if Pd deposition compromised the structural integrity of bR. The photocycle response was observed for all samples, regardless of whether Pd was deposited directly on the protein layer or on the reverse side of the substrate (as a control to assess the effect of light absorption by the Pd film). In both cases, the light‐induced M‐intermediate appeared, with close to 100% conversion from the bR dark state. These results indicate that: 1) the absorption maximum of bR does not shift by solid‐state on surface immobilization and top Pd evaporation. 2) The characteristic photocycle of bR is preserved. As we reported^[^ ^20^, ^31^, ^35^, ^36^ ^]^ before, for bR deposition on other substrates, the photocycle kinetics of bR in dry thin film configuration are slower than in solution, which helps in unambiguously measuring the conversion process. Importantly, the M‐intermediate forms show no change in absorption maximum. The 570 nm absorption maximum of bR is particularly sensitive to conformational changes in the vicinity of the retinal protonated Schiff base, but also to changes in overall secondary and tertiary protein structure.^[^ ^37^, ^38^, ^39^ ^]^ Therefore, the preservation of this maximum absorption implies that any conformational changes of bR upon immobilization and Pd evaporation are insignificant. Furthermore, the preservation of the photocycle following Pd evaporation confirms that the protein layer beneath the optically transparent ≈7 nm Pd film remains functional and structurally intact.

#### 2.6.4 Preservation of Protein IR Signatures Under Pd Top‐Contact Integration

The photocycle measurements provide compelling evidence of functional activity of the bR films in the solid‐state, after top contact evaporation onto them. However, such photochemical responses are limited to a narrow class of photoactive proteins and are not broadly applicable for probing the functional integrity of proteins. A more general method is the reflection mode IR spectroscopy (PEM‐IRRAS) applied to thin films deposited on metal substrate, such as Au.

PEM‐IRRAS offers a sensitive spectral signature of protein conformation through the amide I and II bands, where both peak positions are the manifestation of the protein's overall structural conformation. To examine the influence of metal evaporation on protein integrity, we analyzed multilayers of HSA and bR before and after deposition of a thin Pd layer on top of the multilayer films. A ≈7 nm Pd film was used to ensure sufficient IR transparency while maintaining surface continuity (see Figure S9, Supporting Information).

**Figure 8:**
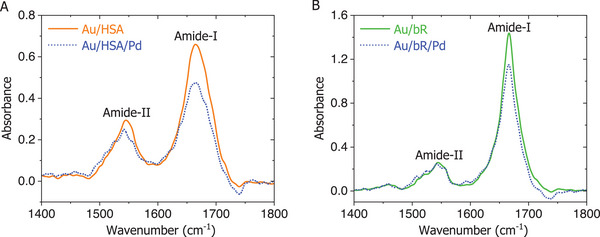
Representative PEM‐IRRAS spectra (averaged over 500 scans; *n* = 500) showing the amide I (≈1670 cm^−1^) and amide II (≈1550 cm^−1^) regions within the 1400–1800 cm^−1^ range for protein films on Au substrates: A) HSA tetralayer and B) bR triple bilayer. Spectra with and without the Pd overlayer are represented by dotted and solid lines, respectively, as indicated in the figure legends. Here, the ordinate shows the ratio of signals from the individual channels, represented as absorbance, more precisely, a pseudo‐absorbance^[^ ^40^ ^]^ (see Section S3.2, Supporting Information).

An intriguing infrared (IR) signature was observed for the protein film following the deposition of such Pd layer: the characteristic amide I (≈1670 cm^−1^), amide II (≈1550 cm^−1^), and C─H stretching (≈2900 cm^−1^) peak positions are closely overlap with those of the without Pd top‐coated protein film, including relatively‐resolved amide bands (see **Figure** **8**; FIgure S10, Supporting Information). This behavior is likely attributable to surface‐enhanced IR absorption^[^ ^41^, ^42^ ^]^ at the protein/Pd interface. In related studies, we are exploring protein‐based surface‐enhanced IR effects. A noticeable shift and broadening of the absorption band ≈3300 cm^−1^ was observed (see Figure S10, Supporting Information), which is primarily attributed to N─H stretching vibrations of the amide A band and/or O─H stretching vibrations from hydrogen‐bonded hydroxyl groups (e.g., from water or side chains such as serine, threonine, and tyrosine). This feature is often qualitatively associated with a hydration layer,^[^ ^43^ ^]^ which is likely to be altered by evaporating the Pd layer on the protein films. These overall results suggest that controlled Pd deposition does not significantly alter the structural integrity of solid‐state protein junctions, regardless of the protein type. Detailed methodologies and sample preparation protocols are provided in the Supporting Information (see Section S3, Supporting Information).

### 2.7 Advantages and Scope of Employing MpD

The MpD chip developed and studied here integrates 16/56 devices, each with an identical structural configuration. The use of an evaporated top electrode and a specific pore size ensures a fixed contact geometry with a precise junction area, reducing uncertainty in junction current variation, which will result otherwise from variations in electrically active area between junctions and experiments. Different from top electrodes that form mechanical contact, contact evaporation yields a larger ratio of electrically active to geometric contact area. Most importantly, the confined protein layer in between the electrode contact retains its functional activity (see Sections 2.6.3 and 2.6.4). The incorporation of two metal contacts, including an evaporated top layer, effectively eliminates the contact resistance in these protein junctions. In a separate study, we systematically addressed the often‐overlooked issue of contact resistance and demonstrated that it becomes negligible in the MpD configuration for proteins.^[^ ^23^ ^]^ Additionally, MpD junctions are well‐suited for low‐temperature transport studies. Unlike other protein junction configurations, MpD ones allow encapsulation and immersion in liquid N_2_ or He, providing a robust platform for investigating transport at cryogenic temperatures (see details in Section 2.6.2) as well as for lab‐to‐lab transfer.

Moreover, MpD can support multiple strategies for protein immobilization, making it highly versatile for studying diverse biomolecules, in this case, both globular and membrane proteins. For example, it can directly couple biomolecules, like the protein HSA, to the Au electrode through cysteine‐mediated Au‐S bonds, or it can do so, as in the case of the membrane protein bR, indirectly through electrostatic interactions using functional linker layers. This dual capability demonstrates that MpD is not limited to a single immobilization mechanism but can be adapted to accommodate different structural and functional classes of biomolecules, and even specific functionalized or charge‐adapted organic molecules. Here, MpD provides a flexible platform for assembling protein films across a variety of systems, with the main requirement being optimization of deposition conditions for each specific biomolecule.

### 2.8 Limitations

Despite its advantages, the MpD configuration has certain limitations compared to protein junctions with soft top electrodes. The requirement for dense protein coverage to achieve transport‐active (electrically non‐shorted) junctions is stricter for MpD devices than for other common configurations, such as junctions with mechanical EGaIn top contact, for which even ≈50% protein coverage suffices for a reasonably high fraction of working junctions.^[^ ^44^ ^]^

The quality of the protein layer significantly impacts junction current distribution, which, in turn, affects the accuracy of thickness‐dependent electron transport characteristics for a given protein. In MpD devices, variations in protein layer thickness cannot be assessed by monitoring impedance‐derived junction capacitance of individual isolated protein junctions. The reason is that the micropores are fabricated at the center of large square bottom electrodes, which are entirely coated with 20 nm of insulating alumina, leaving less than 1% of the surface area exposed.^[^ ^23^ ^]^ This exposed region serves as the active area where the protein layer is added. In the final device configuration, the evaporated top electrode makes contact over a substantial area, effectively forming a junction composed of Au/Al_2_O_3_ (20 nm)/Pd‐Au. In comparison, the area of the central protein‐coated region is negligible (< 1%). Consequently, impedance measurements primarily reflect the capacitive properties of the dielectric alumina, as the low‐dielectric protein layer does not significantly influence the overall capacitance of the single MpD junction. As a result, the protein layer thickness variations cannot be detected from impedance analysis of protein‐based MpD through capacitance measurements, unlike what we did earlier.^[^ ^5^ ^]^ This suggests that junction capacitance is relatively insensitive to local defects and instead reflects the average dielectric behavior across the entire junction area.

In the current MpD device geometry, direct photocurrent measurements cannot be performed because the bottom electrode is fabricated on a Si wafer, while the top electrode is a ≈60 nm thick Pd/Au layer. This design blocks illumination from either side of the junction, thereby preventing light from reaching the embedded protein layer. To still assess the functionality of bR after electrode deposition, we examined its characteristic photocycle optically in a junction with thin enough Pd so that it is partially transparent (see details in Section 2.6.3). These optical studies confirmed that bR retained its light‐responsive properties even after integration into the device structure, which implies that its conformation was not affected.^[^ ^36^, ^45^, ^46^ ^]^ Furthermore, in a separate study using an alternative crosswire device configuration with an evaporated carbon top electrode, we successfully measured a photocurrent response from bR under variable‐wavelength laser excitation.^[^ ^20^ ^]^

## 3 Conclusion

Micropore protein junction‐based devices can be structurally robust with minimal contact resistance^[^ ^23^ ^]^ and consistent electrical response. The robustness of protein‐based MpDs makes it a useful tool for studies aimed at elucidating transport mechanisms in protein junctions. Because in these devices, proteins retain their functionality also after metal evaporation onto them, we foresee that MpDs can become a versatile platform for bioelectronic applications, e.g., bioelectronic sensors, memory devices, and for hybrid bio‐semiconductor interfaces for solid‐state bio/organic electronics, as well as for fundamental studies of electron transport in biomolecules.

## 4 Experimental Section

The multilayered MpD was fabricated by a sequential process. First, a large bottom array of Au electrodes was defined via photolithography, followed by deposition and lift‐off, resulting in patterned electrodes on an insulating silicon wafer. The entire chip was then coated with high‐quality alumina using ALD. A second photolithography step created micropores on the photoresist; the pore window exposed the alumina surface, and the rest of the chip was covered by photoresist. Finally, a combination of dry and wet etching of the alumina/Cr layer, yielded the MpD electrode substrates for protein immobilization. The detailed process is described below.

### Fabrication of MpD—Bottom Electrode Fabrication

The scheme for fabricating the MpD is shown in Scheme 1. Wafers of thick silicon oxide (300 nm) on Si (100) (University Wafer) were used as the base substrate for MpD fabrication. The bottom electrode array was patterned by photolithography using a micro‐writer (see Section S4, Supporting Information, for details). Electron beam evaporation (see Section S5, Supporting Information) was used to deposit a 5 nm chromium (Cr) adhesive layer, followed by a 45 nm gold (Au) layer onto the developed micro‐patterned bottom electrodes. To protect the Au surface from roughening during the subsequent fabrication steps (see Section 2.1), an additional 7 nm Cr layer was deposited on the Au. Subsequently, acetone‐based liftoff (see Section S6, Supporting Information) was performed, resulting in the final patterned bottom electrode stack: [Si (100)/SiO_X_ (300 nm)/Cr (5 nm)/Au (45 nm)/Cr (7 nm)] where the Si (100) wafer was a high‐resistive one.

### Fabrication of MpD—Micropore Fabrication at the Bottom Electrode

The bottom electrode was fully coated with a 20 nm insulating high‐quality (uniform and free of pinholes; see details in Section 2.2) alumina layer using ALD (see Section S7, Supporting Information), as confirmed by high‐resolution‐AFM imaging (Figure S5, Supporting Information). A second round of photolithography was then performed to create micropores at the center of the bottom electrodes, using the protocol described in the Supporting Information (Section S4, Supporting Information), with precise alignment using the micro‐writer. After developing the micropattern, the photoresist exposed a ≈20 *µ*m^2^ window in the alumina layer. The exposed alumina was etched completely using RIE (with CHF_3_ and O_2_ plasma treatments) for 400 seconds (see Section S8, Supporting Information). This process selectively removed the alumina layer, exposing the underlying Cr surface (protective Cr‐layer on top of Au) of the bottom electrode. The choice of specific pore size and its suitability are discussed in Section S9 (Supporting Information). The chip was then treated with Cr etchant (CR‐7 Chrome etch without surfactant, perchloric acid type; FUJIFILM Electronic Materials) for ≈30 s to remove the 7 nm protective Cr layer, followed by immediate rinsing with water and drying with N_2_ gas, and followed by the final liftoff of the photoresist revealed the Au bottom electrode in the micropore, where a 20 *µ*m^2^ window exposed the underlying Au electrode, while the rest of the chip remained insulated by the alumina layer (see Scheme 1). At the boundary of the micropore, residual etched alumina often accumulates as pile‐up material (see the AFM image at the pore boundary, Figures 1 and 2; Figures S2, S4, S6, and S7, Supporting Information). This waste may not be entirely removed during the device cleaning process, and more importantly, it does not adversely affect or interfere with device performance.

### Fabrication of MpD—Protein Layer Preparation in the Micropore

Two types of proteins, HSA, and bR were used to prepare the junctions. The micropore electrode was initially cleaned through sequential solvent sonication for 3 minutes each in acetone, isopropanol (IPA), and Milli‐Q water. Subsequently, the surface of the bottom Au electrode was activated (by removing surface adsorbed contamination /processing residuals) using a base piranha solution (H_2_O: NH_3_: H_2_O_2_ in a 5:1:1 ratio) for 15–20 s at 80 °C. Following activation, the electrode was immediately rinsed with Milli‐Q water and dried under a stream of nitrogen. Then, the micropore electrode was incubated overnight in either a standard HSA solution or, for bR binding, a 4 mg mL^−1^ cysteamine solution in phosphate buffer at pH 7.4. The HSA layer was covalently immobilized onto the bottom gold electrode by gold‐sulfide linkage with its native exposed cysteine groups, while the first bR layer was electrostatically coupled to the cysteamine‐coated linker layer on gold.^[^ ^5^ ^]^ The next protein layer was bound to the first one by in situ EDC treatment to form covalent amide bonds^[^ ^3^ ^]^ for both bR and HSA, without affecting the electrostatic, linker‐assisted immobilization on the substrate. Detailed protocols of the protein layer preparation were described elsewhere.^[^ ^5^, ^23^ ^]^

### Fabrication of MpD—Top Electrode Deposition

A two‐step metal deposition process was used on the protein‐modified micropore, using a precisely aligned shadow mask positioned at the center of the active region of the MpD chip, exposing all protein‐coated micropore windows. Sequential deposition of a 35 nm Pd layer, followed by a 25 nm Au layer, was done in an Angstrom e‐beam evaporator. The MpD chip was mounted on the sample stage, which was placed at 500 mm from the metal source and at a 45° inclination relative to it during deposition, to maximize the probability of only indirect exposure of protein voids to metal vapor. The sample stage temperature was maintained at 18 °C through active cooling to minimize thermal stress on/in the protein layers, and during evaporation, the sample stage was rotated at 10 rpm for uniform deposition. The chamber vacuum was maintained below 10^−7^ torr (< 1.3×10^−4^ Pa). Further experimental details are provided in Supporting Information Section S5 (Supporting Information).

### AFM Characterization

Device fabrication and in situ monitoring were performed using optical microscopy and AFM‐based imaging, primarily with the tapping mode, at various stages of device development. The quality of the immobilized protein layers was assessed, and their thicknesses were determined by AFM scratching using a stiff AFM cantilever (spring constant ≈2 N m^−1^). AFM imaging was done with a *Bruker Nanoscope V Multimode* AFM system, and protein layer scratching was achieved by applying a 150–200 nN contact force.^[^ ^3^, ^5^, ^7^, ^34^ ^]^ Detailed experimental procedures were given elsewhere.^[^ ^5^, ^7^ ^]^ All AFM images were analyzed and processed using *Gwyddion 2.64* software.

### Device Connections for Current/Impedance Response Monitoring

The MpD was interfaced with a probe station using two probe connections (Scheme 2). The top Pd/Au electrode serves as the central electrode, linking all MpDs through a single probe, while the second probe connects individually to each bottom electrode, enabling independent device connections and defining the device's geometric area. By switching between the bottom electrodes, different MpDs were measured. The bottom electrode was covered by the insulated alumina layer. To establish a proper electrical connection, this insulating coating layer was removed by local dry‐etching or scratching from the bottom electrode (see Scheme 2).

The electrical performance of the two probe‐connected devices was assessed using DC and AC voltage sweeps, determining the current and impedance responses of the protein‐functionalized MpDs. For DC measurements, a voltage sweep of ±0.1 V was applied in 1 mV increments, with the bias applied to the central common electrode and the individual bottom electrodes grounded or *vice versa;* this reversal does not influence the current response behavior. For impedance measurements, an AC signal with an amplitude of 10 mV was applied (0 V DC), and the frequency was swept from 1 Hz to 1 MHz, collecting 15–20 data points per decade. The impedance data were analyzed and processed using *Z‐view* 3.2b software (Scribbner Associates). Detailed procedures for the electrical measurements can be found in ref. [5].

### Statistical Analysis

To ensure the reliability and reproducibility of the optimized device structure, multiple control experiments were conducted. Representative device structures were presented through optical microscopy and AFM imaging. The active zone of the MpD, corresponding to the central hole of each device, was quantitatively analyzed. The hole dimensions were obtained by averaging measurements from 10 independent bare devices. Hole depths were determined using the profile extraction tools in Gwyddion software based on 5–10 randomly selected line profiles per device. The resulting values were expressed as mean ± standard deviation (SD), with SD calculated using standard Excel statistical functions. For AFM roughness analysis, data from 256–512 line scans per image were averaged over 2–3 distinct areas per sample and at least 3 independent samples. Roughness values were extracted using the statistical analysis tools in Gwyddion, and the mean and SD were computed in Excel. For *I*–*V* characterization, each single‐junction *I*–*V* curve represents the average of two consecutive voltage sweeps, and the junction current characteristics of protein‐based devices were evaluated by averaging data from 20–25 individual protein junctions. The average *I*–*V* responses were presented with error bars indicating ± SD, estimated using Excel statistical functions. The sample size (n) for each analysis was indicated in the corresponding figure captions. All statistical analyses were performed using Microsoft Excel (Microsoft 365), OriginLab 2024, and Gwyddion (version 2.64).

## Conflict of Interest

The authors declare no conflict of interest.

## Author Contributions

S.B. and D.C. conceptualized device design. S.B., E.M., S.K., and L.T. were responsible for device fabrication and optimization. S.G. provided engineering support for the development of custom‐built instrumentation in the cleanroom. P.L. and S.B. conducted the cryogenic measurements in collaboration with Y.S. S.B. conducted PEM‐IRRAS‐based measurements in collaboration with E. E. S.B., D.C., and A.V., in collaboration with M.S., designed the experiments. S.D. prepared bR‐protein and conducted the photocycle experiments in collaboration with S.B. and M.S. S.B. carried out all preparative steps, device fabrication, characterization, measurements, and data analysis. S.B. wrote the ms. with contributions from all the authors.

## Supporting information



Supporting Information

## Data Availability

The data that support the findings of this study are available from the corresponding author upon reasonable request.
