# Supplementary material for: Hard‐Wired Solid‐State Bioelectronic Micropore Devices: Permanent Metal‐Protein‐Metal Junction Proof‐of‐Concept
Source: Small. 2025 Oct 19;21(49):e06560. doi: 10.1002/smll.202506560 (PMC12696779; doi:10.1002/smll.202506560)
Supplement: Supplementary file 1 — Supporting Information [file SMLL-21-e06560-s001.docx]

Supporting Information

**Hard-wired Solid-state Bioelectronic Micropore Devices****: Permanent Metal-Protein-Metal Junction Proof-of-Concept**

Sudipta Bera,* Eran Mishuk, Ping’an Li, Sourav Das, Sigal Keshet, Sharon Garusi, Leonid Tunik, Eran Edri, Yoram Selzer, Israel Pecht, Ayelet Vilan, Mordechai Sheves,* and David Cahen*

S. Bera, S. Das, M. Sheves, and D. Cahen

Department of Molecular Chemistry and Materials Science

Weizmann Institute of Science

Rehovot 7610001, Israel

Email: [sudipta.bera@weizmann.ac.il](mailto:sudipta.bera.iwhj@gmail.com), [mudi.sheves@weizmann.ac.il](mailto:mudi.sheves@weizmann.ac.il), [david.cahen@weizmann.ac.il](mailto:david.cahen@weizmann.ac.il)

E. Mishuk, S. Keshet, S. Garusi, L. Tunik, and A. Vilan

Department of Chemical Research Support

Weizmann Institute of Science

Rehovot 7610001, Israel

I. Pecht

Department of Immunology and Regenerative Biology

Weizmann Institute of Science

Rehovot 7610001, Israel

P. Li

Department of Chemical and Biological Physics

Weizmann Institute of Sciences

Rehovot 7610001, Israel

E. Edri

Department of Chemical Engineering

Ben Gurion University of the Negev

Beer-Sheva 8410501, Israel

Y. Selzer, P. Li

Department of Chemical Physics, School of Chemistry

Tel Aviv University

Tel Aviv 69978, Israel

**Supporting Information Index**

S1. Cryogenic Setup and Measurement Details (S3)

S2. Sample Preparation and Experimental Setup for Photo-Cycle Measurement (S3-S5)

S2.1 Transmission-Based UV-Vis Setup for Photo-Cycle Analysis (S3)

S2.2 Fabrication of bR Triple Bilayer on Glass (S4)

S2.3 Deposition of Ultra-Thin Pd Top Contact (S4)

S2.4 Photo-Cycle Measurement Protocol (S5)

S3. Photo-elastic Modulated-Infrared Reflection-Absorption Spectroscopy (S5-S6)

S3.1. Sample Preparation (S5-S6)

S3.2. Specification for Data Collection (S6)

S4. Photolithography (S6)

S5. E-beam evaporation (S6-S7)

S6. Lift-off (S7)

S7. Atomic layer deposition (ALD) (S7)

S8. Reactive ion etching (RIE) (S7)

S9. Effect of pore size on device performance (S7-S8)

SI Figures (S9-S15)

**S1. Cryogenic Setup and Measurement Details**

To evaluate the robustness of the protein-based micropore device (MpD), junctions were wire-bonded to a custom-built chip holder (see *Figure S11*). Prior to bonding, stable and transport-active junctions were selected based on their characteristic high-frequency phase response (see *Section 2.5.2* in the main text) and reproducible multi-scan current-voltage (I-V) response behavior. A key challenge was achieving a stable wire-bonding connection to the central top electrode. To enable this, one micropore device was intentionally shorted, and the wire was bonded to the specific contact electrode pad, which is directly electrically connected to the shorted junction, effectively serving as the top-electrode contact.

The wire-bonded junctions were mounted in a cryostat (DE204, *Advanced Research Systems*) equipped with vibration damping and maintained under high vacuum conditions (10^−6^ – 10^−7^ mbar; 10⁻^4^ – 10⁻^5^ Pa). The I-V measurements were conducted using a high-precision source measuring unit (*Keithley 238*) from room temperature (RT, 293 K) down to 10 K. Measurements were performed with a ± 0.1 V sweep, using 1 mV step increments. The experiment began at RT under high vacuum, followed by cooling to 10 K for a second measurement, and subsequent heating back to RT to assess the reversibility of the I-V response with temperature variations. Temperature regulation was achieved via a condenser and cold-finger setup, with precise control maintained by a *Lakeshore 331* controller.

**S2. Sample Preparation and Experimental Setup for *Photocycle* Measurement**

**S2.1. Transmission-Based UV-Vis Setup for *Photocycle* Analysis**

To investigate the *photocycle* dynamics of the sandwiched bacteriorhodopsin (bR) layer, we employed a solid-state UV-Vis transmission setup. For optical transparency, the conventional bottom substrate was replaced with good-quality (extra-smooth) glass, enabling effective light transmission. The top electrode consisted of an ultra-thin evaporated palladium (Pd) layer, designed to mimic the Pd/Au contact in MpD while ensuring sufficient light interaction with the protein layer for spectral detection.

**S2.2. Fabrication of bR Triple-Bilayer on Glass**

Glass substrates of defined dimensions were selected, with an asymmetric marking to distinguish the protein-coated side. The cleaning protocol involved sequential sonication: first in an Alconox solution (5 min), followed by thorough rinsing with Milli-Q water and N_2_ drying. A second cleaning step included sonication in acetone, 2-propanol, and Milli-Q water (3 min each), followed by a hot (80 °C) piranha solution treatment for 5 min (H_2_SO_4_:H_2_O_2_ = 2:1 v/v). The substrates were then extensively rinsed, sonicated in Milli-Q water (2 min), rinsed again, and N_2_-dried. The surface topography of the cleaned, bare glass substrate was checked by tapping mode AFM imaging (see *Figure S9*). For bR immobilization, the surface-chemically resembling Si/SiO_X_ was functionalized with a 3-aminopropyl trimethoxysilane (APTMS) linker.^[1]^ A triple-bilayer of bR was subsequently assembled at the designated region following our previously established protocol.^[1]^

**S2.3. Deposition of Ultra-Thin Pd Top Contact**

To replicate the MpD electrode configuration without compromising optical transmission, a 7 nm Pd layer was deposited via electron beam evaporation (*Angstrom Evaporator*). *Figure S9* shows that Pd forms a smooth, continuous, and uniform layer on glass at any thickness above 5 nm when deposited at a rate of 0.5 Å/s. The estimated roughness of the Pd film, derived from AFM tapping mode imaging, supports the high quality of the ultrathin film on glass. This optimization ensured adequate light penetration while maintaining electrical contact. Control experiments included three different patterns of deposited-Pd layer: (i) 7 nm Pd directly on the bR layer (glass/APTMS/bR/Pd), (ii) Pd on the reverse side of the glass substrate (Pd/glass/APTMS/bR), and (iii) 7 nm Pd on bare glass (glass/Pd) for baseline correction. Deposition parameters are detailed below in *Section S5*.

**S2.4. *Photocycle* Measurement Protocol**

The experiment consisted of three sequential measurements:

1. **Initial Dark Measurement** – A standard UV-Vis scan (300–800 nm) was recorded using an 8540-diode array spectrophotometer (*Hewlett-Packard, USA*).
2. **Light Exposure** – The sample was illuminated with an intense white light source filtered to allow wavelengths >530 nm.
3. **Post-Illumination Dark Measurement** – A delayed (2 min) post-illumination scan was conducted with only the spectrometer illumination.

To prevent stray light interference, the sample stage was shielded with thick aluminum foil, exposing only the measurement area. Baseline correction was performed using the glass/Pd reference substrate. The *photocycle* response was analyzed through differential spectra:

- **∆(LIS-DS)**: Light-induced spectrum (LIS) minus the initial dark spectrum (DS), revealing the M-state signature absorption at ~410 nm wavelength.
- **∆(DS-LIS)**: Final dark spectrum (DS) minus LIS, highlighting the characteristic bR absorption at ~570 nm.

Experiments were conducted on bR triple-bilayers with and without Pd top coating to assess the impact of the electrode on *photocycle* behavior.

**S3. Photo-Elastic Modulated-Infrared Reflection-Absorption Spectroscopy (PEM-IRRAS)**

**S3.1. Sample Preparation**

PEM-IRRAS measurements require a smooth, reflective substrate with an ultrathin film. In this study, gold-coated silicon substrates (Si/Au; rms roughness ~ 0.6 nm) were used for protein film deposition. Substrates were sequentially cleaned in acetone, isopropanol (IPA), and Milli-Q water by bath sonication for 2 minutes each. This was followed by a 30 s base piranha treatment (H_2_O: H_2_O_2_: NH_3_ = 5:1:1) at 80 °C to ensure surface cleanliness and activation; then this surface was rinsed with Milli-Q water and dried under a nitrogen stream. Protein multilayers were prepared on the cleaned Au substrates: a tetralayer of human serum albumin (HSA) and a triple bilayer of bacteriorhodopsin (bR).^[2]^ The layer-by-layer growth was monitored using spectroscopic ellipsometry. Detailed protocols for protein film fabrication are provided in the *Experimental Section, Protein Layer Preparation in the Micropore* (in the main text). For PEM-IRRAS characterization, two sets of each protein film were prepared (a couple of samples for each). One set consisted of pristine protein films on Au substrates, while the other included films coated with the ~7 nm semitransparent Pd layer, deposited at a rate of 0.5 Å/s using the *Angstrom Evaporator* (see *Section S5*).

**S3.2. Specification for Data Collection**

Spectra were acquired using a Thermo Fisher *Nicolet iS50R* spectrometer in photo-elastic modulated IRRAS (PEM-IRRAS) mode, equipped with a liquid nitrogen-cooled MCT/A detector. A ZnSe crystal modulated at 50 kHz with a 0.5 λ retardation at 3000 cm^-1^ was used. Data was collected at 8 cm^-1^ resolution with an 80° incident angle, averaging 500 scans per measurement. All data were collected using a dual-channel sampling operational mode. To reduce noise and moisture contamination, the data were recorded as the ratio of signals from the individual channels, represented here as pseudo-absorbance.^[3]^ Here, the spectra are plotted against ‘pseudo-absorbance’ versus wavenumber for each protein thin film. All experiments were conducted under ambient conditions at room temperature (293 ± 2 K). Spectral processing and analysis were performed using *Omnic 8.1* software.

**S4. Photolithography**

The S1813 positive photoresist was spin-coated onto the substrate at 4000 rpm for 40 seconds, followed by thermal baking at 110°C for 1 minute. The coated silicon substrate was then exposed to a laser with a power density of 250 mJ/cm^2^ using a 10x lens (1 *μ*m resolution, 405 nm wavelength) via a *MicroWriter ML®3* system. The bottom electrode pattern, including alignment marks created in the *layoutEditor* software, was transferred through scanning exposure. The laser-exposed substrate was subsequently developed in MF-319 developer for 80 s, rinsed with deionized water, and dried with a flow of N_2_.

**S5. E-Beam Evaporation**

For bottom electrode fabrication, Chromium (Cr) and gold (Au) layers were deposited with an e-beam evaporation system (*Odem Scientific Applications Ltd.*). The sample was positioned at a height of 300 mm from the metal source, and the chamber vacuum was maintained at <10⁻^7^ mbar (10⁻^5^ Pa) throughout the process. Cr was deposited at a controlled rate of 0.2 Å/s, followed by Au and top Cr deposition at a 0.5 Å/s rate. For the top electrode preparation, Pd and Au layers were deposited sequentially (without breaking vacuum) using a different e-beam evaporation system (*Angstrom Engineering* *Inc, Evovac PDV* platform). The sample was positioned 500 mm above the metal source, and a deposition rate of 1 Å/s was maintained for both metals.

**S6. Liftoff**

The e-beam evaporated substrate underwent liftoff by immersion in acetone for 5 minutes, followed by sonication in acetone for 30 seconds. The substrate was then rinsed with IPA and DI water and dried using a flow of N_2_.

**S7. Atomic Layer Deposition (ALD)**

A *Fiji™* Plasma ALD system was used to deposit a 20 nm insulating alumina layer across the entire substrate. The thermally activated ALD cycle includes pulsing Trimethylaluminum (TMA) and H_2_O, as aluminium and oxygen precursors, respectively, each for a period of 0.06 s and purging for a period of 10 s, with a continuous flow of argon carrier gas. The chamber temperature was maintained at 180°C, and the deposition was conducted for 200 cycles. The alumina layer thickness and process correlation were pre-optimized on blank silicon with a known oxide thickness, and the deposited alumina thickness was verified using ellipsometry and AFM.

**S8. Reactive Ion Etching (RIE)**

The chip was affixed to a clean wafer using wax, treated at 60°C, and then cooled to room temperature. The mounted chip was exposed to CHF_3_ + O_2_ plasma for 600 s in *Omega LPX* Rapier, SPTS, ICP-RIE, etch system. The RIE process was optimized with a CHF_3_ flow rate of 48 sccm and an O_2_ flow rate of 10 sccm, under an applied platen (a special metallic stage for applying power) load power of 50 W and a chamber pressure of 70 mTorr.

**S9. Effect of Pore Size on Device Performance**

We systematically examined how varying the pore diameter impacts device stability and charge transport. Increasing the pore size to ~10 *μ*m severely compromised structural integrity, as larger pores exacerbate undercutting during the final chrome-etchant-based wet etching step (*Section 2.3, Figure 1A, Scheme 1E*). This resulted in fragile devices with extensive shrinkage regions, poor adhesion between the alumina layer and the underlying Au, and frequent delamination of the alumina coating during cleaning. The larger pore area also raised the probability of short-circuited junctions, since the effective protein-coated junction area susceptible to defects scales with the active area, here the pore area. Conversely, reducing the pore size below ~4 *μ*m was limited by both photolithographic resolution and measurement sensitivity. Smaller pores yield reduced junction areas, lowering the signal-to-noise ratio, and for thicker protein layers, the resulting ETp currents often fell below detection limits, hindering systematic studies of length-dependent transport. Balancing these structural and electrical considerations, a pore diameter of ~4 *μ*m was found to be optimal for our device geometry.

B

C

A

**Figure S1.** (**A**) Schematic of the MpD chip unit, comprising 56 integrated devices. The red dotted box highlights the active zone of the chip, where micropores are positioned at the centers of square pads. Each square pad is connected to rectangular pads, which serve as bottom electrodes (within the yellow dotted box); here, the insulating alumina was removed by dry etching, which exposed the bottom Au for electrical contact. (**B**) Enlarged view of the active zone, illustrating the placement of the central top electrode with an array of 56 micropores. (**C**) Single micropore electrode with a square pad area of 100 × 100 *µ*m², containing a 4 × 4 *µ*m² micropore located at the center (black dotted box).

A1

C1

B1

C2

B2

A2

**Figure S2.** Representative (AFM) images (**A1**, **B1**, **C1**) alongside corresponding depth (line) profiles (**A2**, **B2**, **C2**) illustrating pore formation via reactive ion etching (RIE) on ALD-alumina without a protective Cr layer. Pore depth varies depending on the RIE exposure time; (**A1**, **A2**) for 100 seconds with pore depth (7 ± 3 nm), (**B1**, **B2**) for 400 seconds with pore depth (28 ± 2 nm), (**C1**, **C2**) for 600 seconds with pore depth (65 ± 3 nm) [mean ± SD; *n* = 5]. The scattered yellow spots at the pore boundary indicate residual alumina waste, produced by the dry-etching step (see **Section 4.1.2** in the *main text*).

C

B

A

**Figure S3**. Reactive ion-etched Cr surface over the micropore window. (**A**) Tapping-mode atomic force microscopy (AFM) topographic image reveals the central square region (spot area) within the micropore window, indicating no significant etching. (**B**) Corresponding 3D visualization of the surface topography. (**C**) Cross-sectional profile across the spot area around the central region.

A1

C2

B2

A2

C1

B1

**Figure S4**. Tapping mode AFM images (**A1**, **B1**, **C1**) and their line profile cross-section (**A2**, **B2**, **C2**) across the pore area with different extents of Cr wet etching for (**A**) 10 s, (**B**) 20 s, and (**C**) 30 s. The scattered yellow spots at the pore boundary indicate residual alumina waste, produced by the dry-etching step (see ***Experimental Section*** in the *main text*).

**Figure S5.** Tapping mode AFM topographic images of high-quality, uniform (20 nm thick) alumina layer (grown via ALD) on a Si/Cr/Au/Cr substrate, with 0.6 ± 0.2 nm rms surface roughness (mean ± SD; *n* = 10).

B

A

**Figure S6.** (**A**) Tapping mode AFM topography of soft oxygen-plasma treated micropore electrode surface (**B**), high-resolution image at the micropore area with a surface rms roughness > 2 nm (*n* = 5).

B

A

D

C

**Figure S7.** Effect of controlled base piranha treatment on MpD surfaces. (**A**) Prior to piranha treatment, the pore area has a root mean square (rms) roughness of 1.2 ± 0.2 nm. (**B**) Following piranha treatment, the pore area shows decreased rms roughness of 0.6 ± 0.2 nm. (**C**) Depth profile of the piranha-treated MpD reveals a pore depth of 22 ± 2 nm (mean ± SD; *n* = 5). (**D**) Tapping mode AFM topography of the pore area after piranha treatment.

A

B

**Figure S8.** Tapping mode AFM topographic image of (**A**) HSA (**B**) bR monolayer at the pore-region of the MpD. Here, the dark brown to black regions show the direct exposure of the bottom substrate.

C

B

A

**Figure S9.** (**A**) Optical microscope image of a circular, 5 nm thick Pd film, deposited on a glass substrate (scale bar 250 *µ*m). (**A**) the top view of the Pd film, the *yellow* round region at the center, formed by e-beam evaporation through a shadow mask with a 1 mm diameter aperture. The surrounding area corresponds to the bare glass substrate. (**B**), (**C**): AFM tapping-mode images (10 × 10 *µ*m²) of the surface morphology of (**B**) bare glass, with an rms roughness of ~0.4 nm, and (**C**) the Pd-coated region, with an rms roughness of ~0.5 nm (mean, *n* = 3).

A

B

**Figure S10.** Representative PEM-IRRAS spectra in the higher wavenumber region (2800–3500 cm^-1^) for protein films on Au substrates (averaged over 500 scans; *n* = 500): (**A**) HSA tetralayer and (**B**) bR triple bilayer. Spectra with (**Au/protein/Pd**) and without (**Au/protein**) the Pd overlayer are shown as dotted and solid lines, respectively, as indicated in the figure legends.


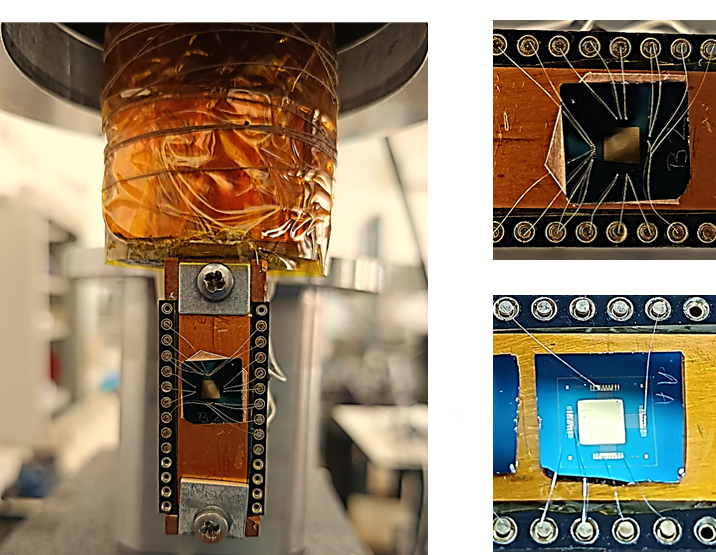


B

A

C

**Figure S11.** Wire-bonded protein-based MpD device. (**A**) Mounted MpD-chip in the cryostat. (**B, C**) Photographs of wire-bonded chips.

**References**

[1] S. Bera, J. A. Fereiro, S. K. Saxena, D. Chryssikos, K. Majhi, T. Bendikov, L. Sepunaru, D. Ehre, M. Tornow, I. Pecht, A. Vilan, M. Sheves, D. Cahen, *J. Am. Chem. Soc.* **2023**, *145*, 24820.

[2] S. Bera, A. Vilan, S. Das, I. Pecht, D. Ehre, M. Sheves, D. Cahen, *True Solid-State Electrical Conduction of Proteins shows them to be Efficient Transport Media*, arXiv, **2025**.

[3] E. A. Monyoncho, V. Zamlynny, T. K. Woo, E. A. Baranova, *Analyst* **2018**, *143*, 2563.
